# BactoBattle: a game-based learning companion for medical bacteriology

**DOI:** 10.1099/acmi.0.000608.v3

**Published:** 2023-06-26

**Authors:** Korakrit Imwattana, Areeya Methawirune, Isara Voralakkulchai, Popchai Ngamskulrungroj

**Affiliations:** ^1^​ Department of Microbiology, Faculty of Medicine Siriraj Hospital, Mahidol University, Bangkok, Thailand; ^2^​ Faculty of Medicine Siriraj Hospital, Mahidol University, Bangkok, Thailand; ^3^​ Department of Surgery, Faculty of Medicine Siriraj Hospital, Mahidol University, Bangkok, Thailand

**Keywords:** antimicrobial resistance, bacteriology, game-based learning

## Abstract

A card game called BactoBattle has been developed to help medical students who have just started learning medical bacteriology to improve their learning efficacy and satisfaction, especially on the topic of antimicrobial resistance. Copies of the game were placed in the students’ study room (approximately 1 set per 12 students) and made available to the students throughout the study period so that they could choose to play the game during their free time if desired. After the study period had ended, the students were asked to complete a questionnaire and a post-test. In total, 33 students completed the questionnaire, and were split into 2 groups: the player group, comprising 12 (36.4 %) students who had played the game, and the non-player group. The player group perceived that they could memorize more knowledge compared to the non-player group and indeed recorded higher post-test scores than the non-player group (10.4 vs 8.3 out of 15 points, *P*=0.031). However, there was no difference in learning motivation (*P*=0.441) or enjoyment (*P*=0.562) between the two groups. A majority of the players said they would continue playing the game after the study period and would recommend the game to other students. In short, the BactoBattle game can be a useful tool to improve the learning efficacy of students, but its effect on learning satisfaction remains unclear.

## Data Summary

The Supplementary Material contains (a) the list of ‘must-know’ bacteria according to the Thai Medical Council criteria, (b) all cards included in the final version of the game, (c) the post-test items and (d) the questionnaire used to evaluate satisfaction and motivation, and is available on Figshare (https://doi.org/10.6084/m9.figshare.22589929) [1]. The complete rulebooks (both Thai and English versions), the copy of the game and the accompanying files are also available on Figshare (https://doi.org/10.6084/m9.figshare.17211860) [2].

**Impact Statement**

This study presents BactoBattle, a game-based learning tool that can be used to teach medical bacteriology and antimicrobial resistance to medical students with little to no prior knowledge on the topic.

## Introduction

Game-based learning is the use of gaming as a tool to enhance and promote learning. This learning method has been proven to help students retain knowledge [3], as well as enhancing their problem-solving and critical-thinking skills, two important attributes of learners in the 21st century [4]. Thus, it has been used in many disciplines, including medical microbiology [5, 6].

To aid in the learning of medical microbiology, several forms of games have been developed, including the well-known online role-playing game Microbe Invader (https://www.microbeinvader.com/), but the most common forms of games are card games or board games [5, 6]. These types of game tend to be based on the use of flashcards, which represent an effective and easy-to-make tool for memorizing recall knowledge [5].

The existing card games developed for learning in medical microbiology can be roughly classified into two groups. The first group targets a specific audience group, e.g. medical students, and teaches specific topics in depth. Some examples include the Bacteria Game and Dawaa [5]. Generally, these games require supervision during play. The second group targets a broader audience and by focusing on basic knowledge that is readily digestible by laypeople, these games can be played without supervision.

At the Faculty of Medicine Siriraj Hospital, Mahidol University, Thailand, the teaching of bacteriology is limited by time constraints. Based on the standard requirements for general practitioners (GPs) set by the Thai Medical Council (TMC) [7], bacteriology lecturers have created a list of 53 ‘must-know’ bacterial species (see Table S1, available in the online version of this article). Typically, students are required to self-study in addition to their allocated class time, a similar situation to that experienced worldwide [8]. Thus, the authors aimed to create a tool to aid in the learning of medical bacteriology.

For a game to be effective as a learning tool, a specific topic is needed as the focus [3]. For this study, antimicrobial resistance was chosen, mostly as it reflects an important global issue today [9], requires the integration of knowledge from various fields and has an underlying theme that can fit into the battle format of a game. Consequently, we present BactoBattle, a card game designed to provide players (medical students) with sufficient knowledge regarding antimicrobial resistance, and that has relatively simple rules and can be played without supervision.

## Methods

### Brief details of the game

This study was based on the game-based theory of learning. Briefly, a game, called BactoBattle, was developed as a tool to aid in learning a specific topic, with the focus being on medical microbiology here. The objectives were to increase learning engagement and motivation among medical students [3]. Antimicrobial resistance was chosen as the main topic of this game, as it is an important but complicated topic [9], and as current teaching methods face challenges in achieving their desired outcomes. Further, the underlying theme of this medical topic are battles between bacteria and antimicrobial agents, which can be readily translated into a battle-style game.

BactoBattle is a two-player turn-based battle card game. The game has three sets of cards: BUG, DRUG and CONDITION. In the game, each player has to play BUG cards (related to diverse bacteria) to inflict damage on their opponent while using DRUG cards (related to diverse antibacterial agents) to defend against the opposing BUG cards. The BUG and DRUG cards can be enhanced by CONDITION cards that serve multiple purposes: developing resistance, increasing bacterial virulence, adding antibacterial combinations, etc. The game ends when a certain amount of damage is inflicted on a player or when all the BUG cards are eliminated from the playing field. The rules of the game were initially developed by the authors and then simplified following feedback from 11 testers (see Acknowledgements) to ensure that the students would be able to play the game independently while ensuring that the game retained scientific accuracy.

The lists of bacteria, antimicrobial agents and mechanisms of antimicrobial resistance were chosen based on the list of must-know knowledge derived from the TMCs [7] and are listed in Table S2. All the cards included in the game reflect common bacteria causing problems in Thailand that general practitioners are expected to know about and be able to manage independently.

### Study population

This study was conducted on second-year medical students in the 2022 academic year. These students study medical microbiology during the second semester (approximately February to March 2023). Prior to this, they had studied basic biology (cell biology and basic genetics) in 2021, but would have little or no basic knowledge of microbiology.

### Game development and the quality-control process

A prototype of the game was developed and given to a group comprising 11 testers (see the Acknowledgements), which included card game enthusiasts, newly graduated general practitioners and other medical students. This group evaluated the game for its relevance and playability. Based on their comments, the first version of the game was created and given to third-year medical students to evaluate its suitability with the targeted audience (the pilot group). After analysis of the pilot group’s feedback and performance, the game was revised and given to the test group.

### Study design

This study was a prospective observational study with minimal interference focused on medical students. Game sets were made available in the students’ study room (estimated 1 set per 12 students), with extra game sets available upon request. All the students were informed about the details of the game as stated above by one of the study authors (K.I.) at the beginning of the study period. Each student could then freely choose to inspect and/or play the game as desired.

At the end of the study period (approximately 1 month after the beginning of the study), all students were asked to complete a self-report questionnaire, focusing on their competence in the subject area and overall learning satisfaction. For students who had not played the game, the questionnaire also focused on their reasons for not playing and any alternative learning tools that they may be interested in. After completing the questionnaire, each student was then asked to complete a post-test that evaluated their knowledge of the three main topics of the game: medical bacteria, antibacterial agents and antimicrobial resistance mechanisms. The English translation of the post-test is available as File S1. As the students had little or no prior knowledge of bacteriology before the study period, their self-reporting of their academic achievements (e.g. past outstanding or unsatisfactory grades) was used to compare their base competence [10].

### Statistical analysis

Statistical analysis was performed using an online tool available at the Social Science Statistics website (https://www.socscistatistics.com/). The normality of the post-test scores was evaluated using Kolmogorov–Smirnov test (*P*=0.315). A two-sample *t*-test was used to compare the post-test scores. The Mann–Whitney U test was used to compare the self-reporting parameters: perceived competence and learning satisfaction. A *P*-value of <0.05 was used to define statistical significance.

## Results

### Study population

In total, 33 students completed the questionnaire and post-test, of whom 12 (36.4 %) had played the game during the study period. There were no differences between the number of students with previous outstanding (75.0 vs 62.5 %, *P*=0.590) and unsatisfactory grades (37.5 vs 66.7 %, *P*=0.229) between the player and non-player groups, respectively.

### Evaluation of the learning efficiency

The learning efficacy was evaluated using two methods: students’ self-evaluation and their post-test scores. For self-evaluation, the player group perceived that they had higher competence compared to the non-player group in all three topics: bacteria (3.6 vs 2.6, *P*=0.012), antimicrobial agents (3.3 vs 2.1, *P*=0.008) and resistance mechanisms (2.7 vs 1.9, *P*=0.046).

For the post-test scores, the player group scored higher than the non-player group (10.4 vs 8.3, *P*=0.031). When separated by topic, the difference in the post-test scores was significant and most pronounced in the topic of resistance mechanisms (3.4 vs 2.5, *P*=0.027), while the differences in the bacteria (3.6 vs 3.1, *P*=0.092) and antibacterial agents (3.4 vs 2.7, *P*=0.064) topics were not statistically significant.

### Evaluation of learning satisfaction

The player group gave the BactoBattle game high scores regarding their enjoyment in playing the game (3.8 out of 5) and the game’s effect on their learning motivation (3.7 out of 5). However, there was no difference in the overall learning enjoyment (*P*=0.441) and motivation scores (*P*=0.562) between the player and non-player groups.

A large proportion of players (11; 91.7 %) said they would continue playing the game after the study period. Most of them (11; 91.7 %) would also recommend the game to other students.

### Reasons for not playing the game

As the number of players was relatively small and in the clear minority, the non-players were asked for their reasons for not playing the game. The most common reason was a lack of time (66.7 %), followed by a scarcity of game sets available (38.1 %) and the perceived inappropriateness of the study site (14.3 %).

### Other learning tools

The students who had not played the game were also asked about what other types of learning tools they would have preferred. The most common answers were: (1) regular flash cards (76.2 %), (2) self-assessment quizzes (66.7 %) and (3) other game formats (42.9 %).

## Discussion

To assist the medical students with their self-study on the topic of antimicrobial resistance, a flashcard-based game, BactoBattle, was developed and evaluated. The two main objectives were to improve learning efficacy and increase student satisfaction with learning the topic [3]. This study investigated the efficacy of the game in improving second-year medical students’ knowledge of bacteriology.

The students who had played the game perceived that they had higher competence than those who had not played the game and indeed had higher post-test scores, with the largest difference noted for the topic of resistance mechanisms. This suggests that the BactoBattle game can indeed help students to learn basic bacteriology, especially the topic of antimicrobial resistance. The highest score difference being on the topic of resistance mechanisms might be somewhat expected, as this topic involves critical thinking rather than just simple memorization. In the game, the player has to respond to the opponent’s DRUG card and adjust their resistance mechanism. This process would stimulate a higher level of learning [11]. The players also scored marginally higher in the topics of bacteria and antibacterial agents, suggesting that the game mechanics may also serve as regular memory prompts, like flash cards, to help with basic memory.

The effect of the BactoBattle game on learning satisfaction, however, was unclear. Most of the parameters evaluated were not significantly different between the player and non-player groups. Admittedly, the study period was only 1 month in both groups, which may not have been sufficient for the game to have an effect on student satisfaction. A follow-up study with a longer study period is needed to clarify this point.

There were several limitations of this study to note. First, the study was cross-sectional in nature. To maintain the confidentiality of the participants, personal details (except for the students’ previous academic achievements) were not collected in the questionnaire. Thus, individual progression could not be tracked. As the personal details were not recorded, the post-test performance could not be linked to external matrices. Instead, the students’ self-reporting of academic achievements was used as a broad marker of their academic competence. This has been reported by several studies to be an accurate representation of actual overall performance [10], but not a subject-specific one. Second, the study was observational in nature, in order to maintain the ‘normal’ learning environment. It was thus not possible to confirm that the players did indeed play the game as they claimed, and nor could the frequency of play be determined. Finally, the number of players was relatively small in this study. This was partially expected, given that this was the first time the game had been introduced into the learning environment, with little evidence to support its effectiveness prior to this study. Thus the power of this study is limited, although it provides a preliminary insight into how game-based learning can be helpful in this setting and some encouraging results to suggest that further development of the game could increase its effectiveness. From the results of this study, it is expected that the proportion of players will increase in future classes based on the recommendations of the current students. Further, the future class schedule could be designed to allow more time for students to play the game.

## Conclusion

BactoBattle is a card-based learning game that students can play during their free time. The use of the BactoBattle game to aid the learning of medical bacteriology can improve student’s perception of their competence in this field and their learning efficiency. However, the effects of the game on students’ learning enjoyment and learning satisfaction were unclear.

## Supplementary Data

Supplementary material 1
