# Supplementary material for: BactoBattle: a game-based learning companion for medical bacteriology
Source: Access Microbiol. 2023 Jun 26;5(6):acmi000608.v3. doi: 10.1099/acmi.0.000608.v3 (PMC10323792; doi:10.1099/acmi.0.000608.v3)
Supplement: Supplementary material 1 [file acmi-5-608.v3-s001.pdf]

## Supplementary Material

**BactoBattle: a learning companion for medical bacteriology**

### A. List of 'must-know' bacteria

| Gram positive bacteria | Gram negative bacteria | Other bacteria |
|---|---|---|
| *Enterococcus faecalis* | *Acinetobacter baumannii* | *Chlamydia trachomatis* |
| *Enterococcus faecium* | *Aeromonas hydrophila* | *Chlamydophila pneumoniae* |
| *Staphylococcus aureus* | *Bordetella pertussis* | *Mycoplasma pneumoniae* |
| *Streptococcus agalactiae* | *Burkholderia pseudomallei* | *Orientia tsutsugamushi* |
| *Streptococcus pneumoniae* | *Campylobacter* spp. | *Leptospira interrogans* |
| *Streptococcus pyogenes* | *Enterobacter* spp. | *Treponema pallidum* |
| *Streptococcus suis* | *Escherichia coli* | |
| Viridans streptococci | *Fusobacterium nucleatum* | |
| *Moraxella catarrhalis* | *Haemophilus ducreyi* | |
| *Neisseria gonorrhoeae* | *Haemophilus influenzae* | |
| *Neisseria meningitidis* | *Helicobacter pylori* | |
| *Actinomyces* spp. | *Klebsiella pneumoniae* | |
| *Bacillus anthracis* | *Legionella pneumophila* | |
| *Bacillus cereus* | Non typhoidal *Salmonella* | |
| *Clostridiodes difficile* | *Pseudomonas aeruginosa* | |
| *Clostridium botulinum* | *Salmonella* Typhi | |
| *Clostridium perfringens* | *Shigella* spp. | |
| *Clostridium tetani* | *Vibrio cholerae* | |
| *Corynebacterium diphtheriae* | *Vibrio parahaemolyticus* | |
| *Cutibacterium acnes* | *Vibrio vulnificus* | |
| *Listeria monocytogenes* | | |
| *Mycobacterium avium* | | |
| *Mycobacterium leprae* | | |
| *Mycobacterium tuberculosis* | | |
| *Nocardia* spp. | | |

**B. List of BactoBattle cards**

| BUG cards | DRUG cards | CONDITION cards |
|---|---|---|
| **Gram positive** | **Aminoglycosides** | **Resistance mechanisms** |
| *Bacillus anthracis* | Amikacin | 30s ribosome variation |
| *Clostridioides difficile* | Gentamicin | 50s ribosome variation |
| *Clostridium perfringens* | **Carbapenems** | ESBL |
| *Corynebacterium diphtheriae* | Ertapenem | Gyrase mutation |
| *Enterococcus faecalis* | Imipenem | *mecA* gene |
| *Staphylococcus aureus* | Meropenem | Multidrug efflux pump |
| *Streptococcus pneumoniae* | **Cephalosporins/cephamycins** | Penicillinase |
| *Streptococcus pyogenes* | Cefazolin | *vanA* gene |
| Viridans streptococci | Cefepime | **Others** |
| **Gram negative** | Cefoxitin | Horizontal gene transfer |
| *Acinetobacter baumannii* | Ceftriaxone | Hypermutation condition |
| *Escherichia coli* | **Fluoroquinolones** | Hyper-virulence |
| *Haemophilus influenzae* | Ciprofloxacin | Re-infection |
| *Klebsiella pneumoniae* | Norfloxacin | Beta-lactamase inhibitor |
| *Neisseria meningitidis* | **Glycopeptides** | Dosage adjustment |
| *Pseudomonas aeruginosa* | Vancomycin | Drug combination |
| *Salmonella* Typhi | **$MLS_B$** | Drug utilization evaluation |
| *Vibrio parahaemolyticus* | Azithromycin | Enhanced bactericide |
| **Obligate intracellular** | Clindamycin | Synergistic combination |
| *Chlamydia trachomatis* | Erythromycin | Paracetamol |
| *Mycoplasma pneumoniae* | **Penicillins** | |
| *Orientia tsutsugamuchi* | Amoxicillin | |
| | Dicloxacillin | |
| | Penicillin | |
| | Piperacillin | |
| | **Polymyxins** | |
| | Colistin | |
| | **Tetracyclines** | |
| | Doxycycline | |

**C. Posttest questions**

Note: The options for each question below did not represent the actual arrangement.

Question topics; B Bacteria
D Drugs (antimicrobial agents)
R Resistance mechanisms

[B 1] Which of the following is an obligate intracellular pathogen?

1. *Enterococcus faecalis*
2. *Orientia tsutsugamushi* [Key]
3. *Vibrio parahaemolyticus*
4. *Acinetobacter baumannii*

[p = 0.70; point biserial = 0.58]

[B 2] Which of the following is a Gram-positive bacterium?

1. *Escherichia coli*
2. *Bacillus anthracis* [Key]
3. *Klebsiella pneumoniae*
4. *Chlamydia trachomatis*

[p = 0.94; point biserial = 0.17]

[B 3] Which of the following can cause a severe disease that progresses rapidly?

1. *Clostridioides difficile*
2. *Streptococcus pyogenes* [Key]
3. *Acinetobacter baumannii*
4. *Mycoplasma pneumoniae*

[p = 0.30; point biserial = 0.28]

[B 4] Which of the following is well-known for its resistance?

1. *Bacillus anthracis*
2. *Neisseria meningitidis*
3. *Klebsiella pneumoniae* [Key]
4. *Streptococcus pyogenes*

[p = 0.79; point biserial = 0.48]

[B 5] Which of the following is well-known for its ability to produce biofilms?

1. *Bacillus anthracis*
2. Viridans streptococci [Key]
3. *Streptococcus pyogenes*
4. *Streptococcus pneumoniae*

[p = 0.55; point biserial = 0.41]

[D 1] Which of the following penicillins can kill *Staphylococcus aureus*?

1. Amoxicillin
2. Penicillin G
3. Piperacillin
4. Dicloxacillin [Key]

[p = 0.67; point biserial = 0.36]

[D 2] Which of the following can be given with penicillins for a synergistic effect?

1. Amikacin [Key]
2. Imipenem
3. Vancomycin
4. Azithromycin

[p = 0.36; point biserial = 0.55]

[D 3] Which of the following is a drug of choice for *Clostridioides difficile* infection?

1. Colistin
2. Cefazolin
3. Gentamicin
4. Vancomycin [Key]

[p = 0.61; point biserial = 0.57]

[D 4] Which of the following is a drug of choice for *Salmonella* Typhi infection?

1. Cefoxitin
2. Cefazolin
3. Cefepime
4. Ceftriaxone [Key]

[p = 0.70; point biserial = 0.56]

[D 5] Which of the following is effective against *Mycoplasma pneumoniae* infection?

1. Cefoxitin
2. Amikacin
3. Imipenem
4. Erythromycin [Key]

[p = 0.64; point biserial = 0.50]

[R 1] Gyrase mutation is the mechanism of resistance to which antimicrobial class?

1. Polymyxins
2. Carbapenems
3. Aminoglycosides
4. Fluoroquinolones [Key]

[p = 0.52; point biserial = 0.70]

[R 2] Which mechanism confers tetracycline resistance?

1. 30S ribosome alteration [Key]
2. 50S ribosome alteration

[p = 0.94; point biserial = 0.30]

[R 3] The *vanA* gene is associated with the resistance of which antimicrobial class?

1. Penicillins
2. Polymyxins
3. Glycopeptides [Key]
4. Cephalosporins

[p = 0.48; point biserial = 0.57]

[R 4] Which of the following may share a resistance mechanism with azithromycin?

1. Amikacin
2. Gentamicin
3. Clindamycin [Key]
4. Vancomycin

[p = 0.73; point biserial = 0.35]

[R 5] ESBL is ineffective against which antimicrobial agent?

1. Cefoxitin [Key]
2. Cefazolin
3. Cefepime
4. Ceftriaxone

[p = 0.18; point biserial = 0.40]

**D. Questionnaire**

Part 1: Student's past academic achievement.

Please answer the following yes/no questions.

1. Until the present day, have you ever received an outstanding "O" grade in any subjects?
2. Until the present day, have you ever received an unsatisfactory "U" grade or had to retake an exam in any subjects?

Part 2: Student's participation.

1. Did you play BactoBattle game during the study period? [YES/NO]
2. If you played the game, who were you playing the game with?
    a. One individual
    b. Up to 5 individuals
    c. Up to 10 individuals
    d. More than 10 individuals

Part 3: Self-report parameters.

For the following questions please rank yourself from 0 – 5, where 0 means least likely and 5 means most likely.

1. I enjoy learning medical bacteriology.
2. I want to study more about bacteriology outside of the classroom.
3. I can remember medical bacteria well.
4. I can remember antimicrobial agents well.
5. I can remember the mechanisms of antimicrobial resistance well.

For players, please answer the following additional questions.

6. I enjoy playing BactoBattle.
7. I feel like BactoBattle makes me want to learn more about bacteriology.
